# Evolutionary Analysis of Pre-S/S Mutations in HBeAg-Negative Chronic Hepatitis B With HBsAg < 100 IU/ml

**DOI:** 10.3389/fpubh.2021.633792

**Published:** 2021-04-26

**Authors:** Yingping Wu, Zhiqiang Zhu, Jianyong Wu, Wenzi Bi, Wei Xu, Xiaoping Xia, Dongsheng Han

**Affiliations:** ^1^Department of Clinical Laboratory, Fourth Affiliated Hospital of Zhejiang University School of Medicine, Jinhua, China; ^2^Clinical Medical Examination Center, Northern Jiangsu People's Hospital, Yangzhou, China

**Keywords:** inactive HBsAg carrier, reactivation phase, genome diversity, pre-S/S region mutation, selective pressure

## Abstract

**Background:** Hepatitis B surface antigen (HBsAg) and viral load are important clinical indicators for antiviral therapy. Few studies have evaluated viral sequence biomarkers predicting the risk of hepatocellular carcinoma (HCC) in the stage, which show a low serological response (HBsAg < 100 IU/ml) and high viral levels (HBV DNA > 2,000 IU/ml). This study aims to determine the trend of the biological prevalence within the pre-S/S regions of special model of inactive CHB infection.

**Methods:** We used Sanger sequencing, quantitative HBV serology (HBeAg and HBsAg), and liver function index to identify whether HBV genome sequences are associated with long-term risk of further HCC progression in special inactive CHB infection.

**Results:** HBV sequencing analysis of 28 CHB patients with special infectious pattern showed higher genetic diversity among four opening reading frames (ORFs) (*p* < 0.001). However, dN/dS ratios of HBsAg and pre-C/C regions in the experimental group showed no significantly different from those in the HCC group (*p* = 0.06), while significantly lower in polymerase and HBxAg regions of the experimental group (*p* < 0.001). In addition, seven positively selected sites were identified in pre-S1, five in pre-S2, and four in S, in which five sites (128H/135Q/135R/139L/141P) were among “α” determinant.

**Conclusions:** These mutations in the pre-S/S region might be associated with the HCC phenotype of low HBsAg expression, with the P region possibly impacting high viral loads. Increased viral diversity across the HBV genome is also associated with low levels of HBsAg. The cumulative evolutionary changes in the HBV pre-S/S regions shows that facilitate immune evasion should be monitored individually. Due to the similarity of evolutionary characteristics in HCC, low serological responses and high viremia may be associated with the risk of further disease progression.

## Introduction

Hepatitis B virus (HBV) infection has a wide spectrum of clinical manifestations ranging from an asymptomatic carrier state (immunotolerant state) to acute or chronic hepatitis, with progression to severe liver disease (1). Although a valuable therapies are highly effective at controlling viral replication, they often involve life-long treatment because infections with HBsAg seroconversion are very rarely cured.

Previous studies have reported that the serum HBsAg level is related to intrahepatic covalently closed circular DNA (cccDNA) (2). Lowering of serum HBsAg to an undetectable level may indicate that intrahepatic cccDNA has been eradicated, which is known as a “functional cure”; however, this state is difficult to achieve through current antiviral approaches (3, 4). At the same time, serum HBV DNA levels may indirectly reflect immunological control of HBV infection independent of the HBV DNA viral load, which is considered to represent viral replication activity (5). In the present study, we focused on a special infectious pattern between the low replicative phase (LR, also referred to as the “inactive HBsAg carrier” state) and the reactivation phase [RA, previously also referred to as “HBeAg-negative/anti-HBe positive chronic hepatitis B” (CHB)] that is characterized by a low serological response (HBsAg < 100 IU/ml) and high viral levels (HBV DNA > 2,000 IU/ml), persistently normal aminotransferase levels and mild inflammation and minimal fibrosis in the liver; this pattern is herein referred to as LR/RA CHB.

Recent studies have demonstrated that serum HBsAg levels are a highly predictive factor of a sustained outcomes due to the nature and strength of the host immune response against HBV (6). Monitoring HBsAg levels may aid in tracking the natural history of the disease and in predicting the response to antiviral treatment and natural immune clearance (7). Due to low HBsAg levels, the incidence of HBsAg loss in high genotypes B, C, and D patients in CHB is linked with a lower risk of HCC (8). In this study, we evaluated whether HBsAg level below 100 IU/ml can predict a lower risk for HCC in patients with HBV DNA > 2,000 IU/ml.

The diversity of the HBV genome is important for individualized therapies for CHB patients. Compared with HCC, the HBV genome, including the P/X/S/C regions, exhibits greater diversity, and positive selective pressure is observed within the pre-S/S region. These mutations have been shown to occur within the “α” determinant region of the S region associated with HCC. Nevertheless, the interaction between pre-S/S region mutations and host immunity in LR/RA CHB is still unknown. The aim of the present study was to examine whether this infectious module from LR to RA is related to the risk of developing HCC. We clarify the association between the evolutionary diversity of the HBV genome (especially pre-S/S mutations) and disease stage under LR/RA CHB, screening for new molecular markers of HCC. Furthermore, this study provides novel insight into low HBsAg levels.

## Materials and Methods

### Patients

Twenty-eight HBeAg-negative patients with chronic hepatitis B from the Fourth Affiliated Hospital of Zhejiang University were achieved the inclusion criteria, that is, presence of HBsAg (<100 IU/ml) for at least 6 months, high HBV DNA levels (>2,000 IU/ml), elevated or normal serum alanine aminotransferase (ALT)/aspartate transaminase (AST) levels (upper limit of normal, 50 U/L), and no signs of human immuno-deficiency virus, hepatitis C virus, or hepatitis virus coinfection and other liver disease. Patients who received nucleotide/nucleoside analog treatment (i.e., lamivudine, adefovir dipivoxil, telbivudine, or entecavir), and alcohol or drug abuse would be excluded. In our study, all of 28 CHB patients were assigned to an experimental group and the other 45 HBV-related HCC patients (reference to NCBI database) were identified as the control group. Written informed consent was obtained from all patients, and the study was approved by the Ethics Committee of Fourth Affiliated Hospital of Zhejiang University School of Medicine in accordance with the Helsinki Declaration.

### Extraction of Viral DNA and Genome-Length PCR

HBV DNA was extracted from 200 μl of serum samples from patients with RA CHB using QIAamp DNA Blood Mini Kit (QIAGEN, Hilden, Germany) according to the manufacturer's instructions. We performed semi-nested PCR to amplify four partially overlapping DNA fragments that encompass the complete HBV genome sequence using HotStarTaq plus DNA polymerase (QIAGEN, Hilden, Germany), primers, and conditions as previously described. The PCR products were then sequenced bidirectionally with second-round primers using an Applied Biosystems 3730XL (Applied Biosystems, CA, USA). The full-length HBV genomic DNA sequence was amplified by an Applied Biosystems 2720 thermal cycler (Applied Biosystems, CA, USA) in a 25 μl volume containing 1 μl of HBV DNA template, 0.5 μl of Taq DNA polymerase (5 U/μl), 2.5 μl of 10x Taq buffer, 2 μl of 25 mM MgCl_2_, 0.5 μl of dNTPs (10 mM each), and 1 μl of each primer. The primers were designed according to the corresponding reference (9). The total genomes of 28 viral strains from the 28 patients were sequenced.

### Phylogenetic Analysis

DNAstar software (DNASTAR Inc., Madison, WI, USA) was used to assemble the sequences into a complete HBV genome sequence, and all mutations were refined manually. Phylogenetic trees were reconstructed from either the full-length HBV genomes or concatenated nucleotide alignments (four protein-coding: Polymerase, LHBsAg, HBxAg, and PreC/C). Mafft v7.0.17 was used for alignment of sequences (10), and ModelFinder was used for nucleotide replacement model selection (11). According to the results, GTR+I+F+G4 model was used for MrBayes tree construction in MRBayes v3.2.7 with 10-milion generation (12).

### Sequence Diversity

Sequence diversity was calculated using Shannon entropy (Sn) by R software, which measures the diversity of the number of haplotypes and their frequencies. The diversity of each nucleotide position (nt1 to 3,215) was calculated as the Shannon entropy [Sn = *i*∑ [A, T, C, G, -] (pilnpi)/lnN], where pi represents the relative frequency of nucleotides or deletion at this position and *N* the total number of sequences (13). The illustrating diversity (**Figure 2**) was evaluated in MEGA 7.0 with three parameters (14), the mean genetic distance (d), the number of synonymous substitutions per synonymous site (dS), and the number of non-synonymous substitutions per non-synonymous site (dN); it was generated using the Circos visualization tool (15). All samples were compared with the HCC reference indicated above to identify variations in ORFs at the amino acid level.

### Analysis of Selection Pressure and Positive Selection

We used the dN/dS ratio, a measure of selection, to assess the selection pressure acting on a lineage of HBV. The dN, dS, and dN/dS ratio were estimated using the codeML model in PAML4 (16). Lineage-specific mean values were estimated with concatenated alignments of all orthologs. The KaKs calculator was used to calculate dN/dS in a sliding window and scale (http://www.bacteriamuseum.org/SWAAP/SwaapPage.htm) using the Nei-Gojobori distance estimation method (window length, 57 nt; window step, 6 nt). The direction of selection pressure was determined by measuring the variable ω, representing non-synonymous/synonymous substitution ratios (ω = dN/dS) at each codon site, with values of ω < 1, ω = 1, and ω > 1 indicating purifying selection, neutral evolution, and positive selection, respectively. The extent of positive selection was analyzed using a site model employing two different pairs of models (M1/M2, M7/M8). Model M1 assumes negative selection and neutral evolution; model M2 assumes an additional level of positive selection. The M7 and M8 model pair assumes beta distributions for ω among sites, providing a sensitive test for positive selection. Likelihood ratio tests were utilized to compare the nested models, and empirical Bayes methodology was applied to identify the amino acid sites under positive selection due to a more reliable posterior probability calculation for small datasets (17). Finally, PyMOL 2.3 software was used to show amino acid variants in HBV epitopes with a three-dimensional conformation.

### Statistical Analysis

The distribution of point mutations within the pre-S/S regions at amino acid substitutions within the large HBsAg antigen in the experimental group was evaluated with a Chi-square test using SPSS 19.0. Sequence complexity or diversity was analyzed with Student's *t*-test, and *p*-values of <0.05 were considered significant.

## Results

### Cohort Characteristics

HBeAg-negative CHB patients with coexisting low HBsAg levels (<100 IU/ml) and high HBV DNA levels (>2,000 IU/ml) (*n* = 28) exhibited similar clinical profiles, including their mean age, sex, alanine aminotransferase (ALT) levels, aspartate transaminase (AST) levels, hepatitis B surface antigen (HBsAg), and hepatitis B DNA (HBV DNA) (Supplementary Table 1, Supporting Information). The experimental group was in the reactivation phase. HBV-related HCC sequences were present in the control group (HCC group). The HBV DNA level in the experimental group was significantly higher than that in the regular reactivation phase (>2,000 IU/ml), but with low levels of HBsAg (<100 IU/ml) and normal ALT, which are not common. In total, 28 full-length HBV genomes were analyzed for viral diversity, phylogenetic divergence, selection pressure, and positive selection.

### Phylogenetic Analysis

**Figure 1:**
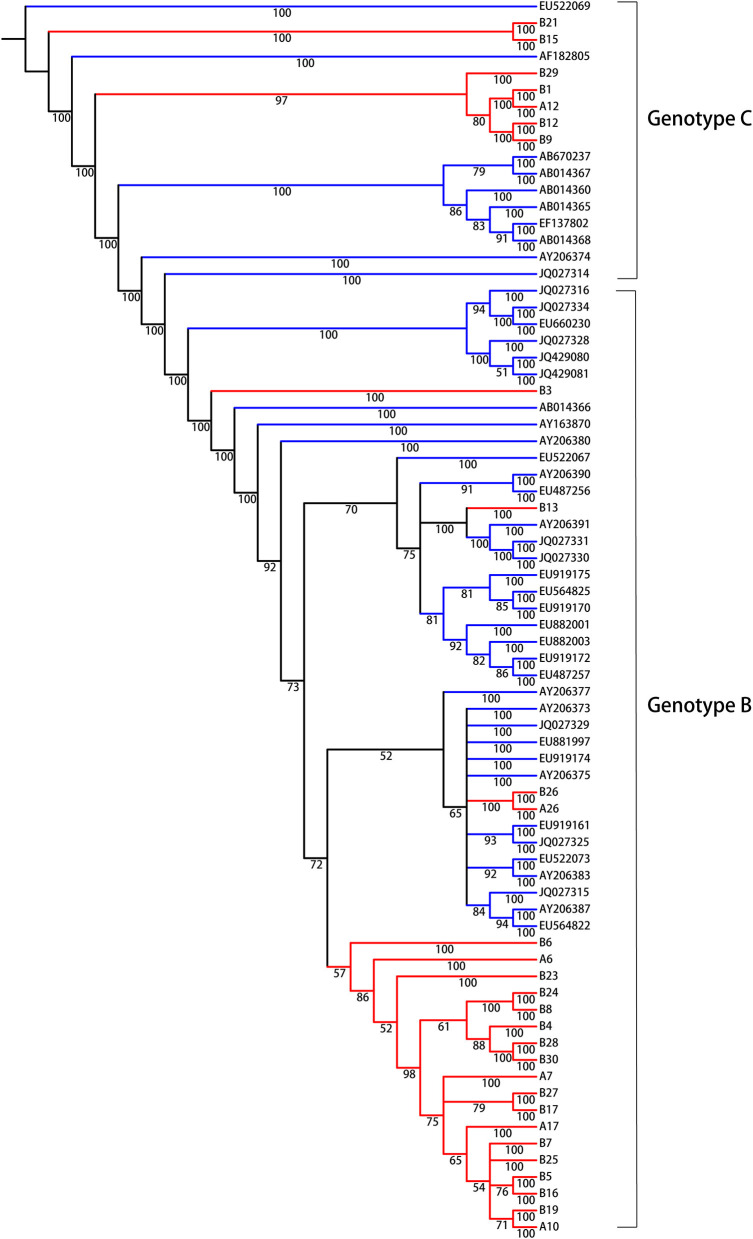
Bayesian consensus phylogeny of HBV isolates for genotyping analysis. The phylogram represents a consensus of 28 HBV sequences from model of inactive CHB infection. Phylogenetic trees were constructed for sequences from both the experimental group (red) and the reference sequences of HBV whole genome downloaded from NCBI (green) with a Bayesian method. Posterior probabilities exceeding 0.5 are shown in the branches. From the phylogenetic analysis, 7 viral strains belong to genotype C and 21 strains are genotype B.

We were interested in identifying molecular evolutionary characteristics specific to LR/RA CHB, and employed population sequencing to analyze the relationship between LR CHB and HCC. The entire HBV whole genome from HCC patients were to be control group and the accession numbers are showed as supplementary data (Supplementary Table 2, Supporting Information). Due to different prognosis risks among HBV genotypes, the HCC group was divided into genotypes B and C; the experimental group was similarly divided. The analytical data indicated the presence of HBV genotypes B and C in the experimental group (Figure 1). In general, all sequences clustered together according to genotype, and there were 22 strains (75%) of genotype B and 6 strains (25%) of genotype C. No other genotypes were detected. The identified HBV genotypes of group I (A6, A7, A10, A12, A17, A26, HBV DNA, >2,000 IU/ml and <20,000 IU/ml) and group II (B1, B3, B4, B5, B6, B7, B8, B9, B13, B15, B16, B17, B19, B21, B23, B24, B25, B26, B27, B28, B29, B30, HBV DNA, >20,000 IU/ml) are the main common genotypes in China.

### Diversity of HBV Sequences in RA CHB

**Figure 2:**
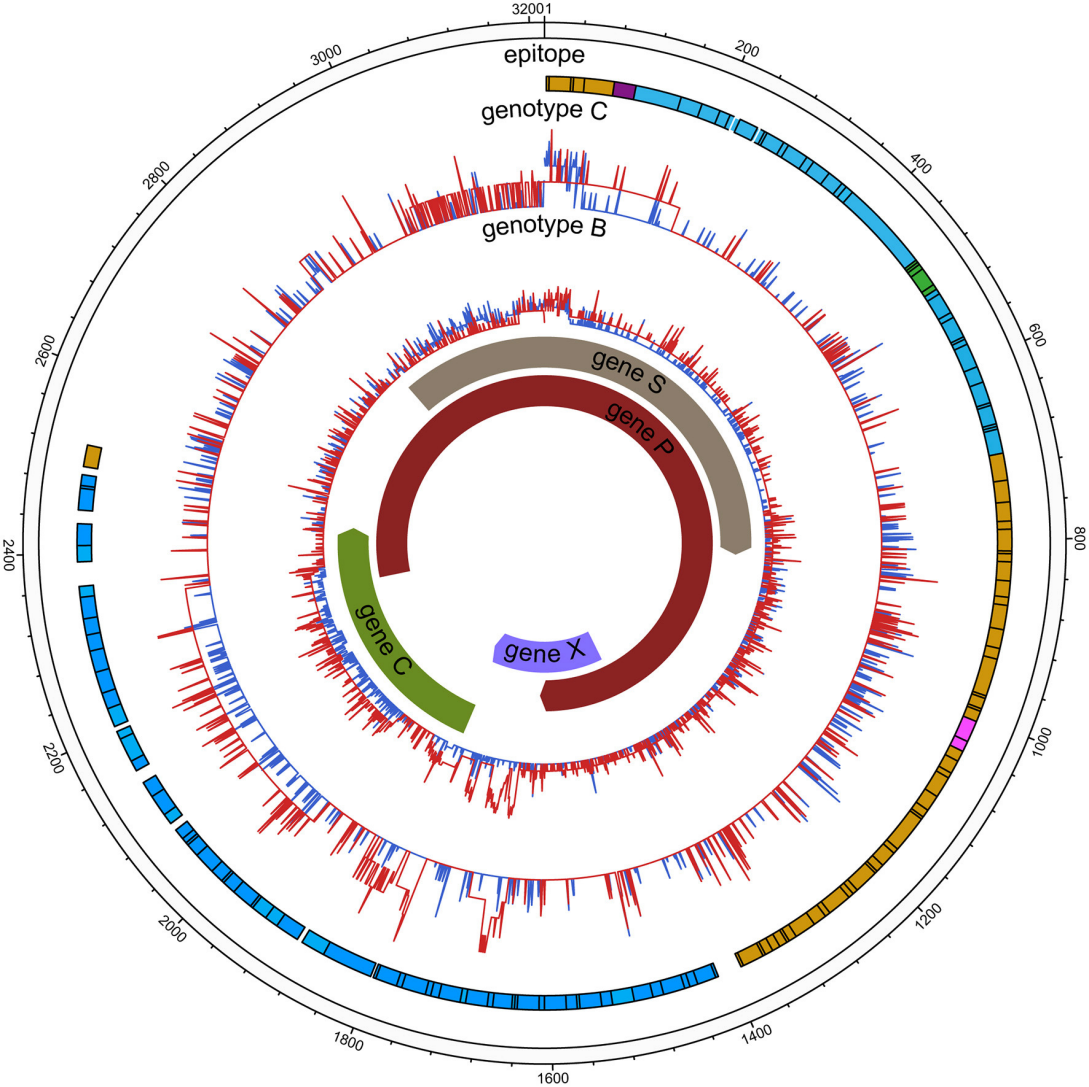
HBV nucleic acid complexity for the experimental group and control group (HCC). The colored bars indicate the complexity of each nucleotide for the experimental group (red lines) and control group (blue lines) for the full-length HBV genome. The insertions were discarded. Epitope distribution is as follows: B cell epitopes (purple), HLAI T cell epitopes (yellow), HLA II T cell epitopes (carnation), overlap of two types of epitopes (green), overlap of three types of epitopes (blue).

**Figure 3:**
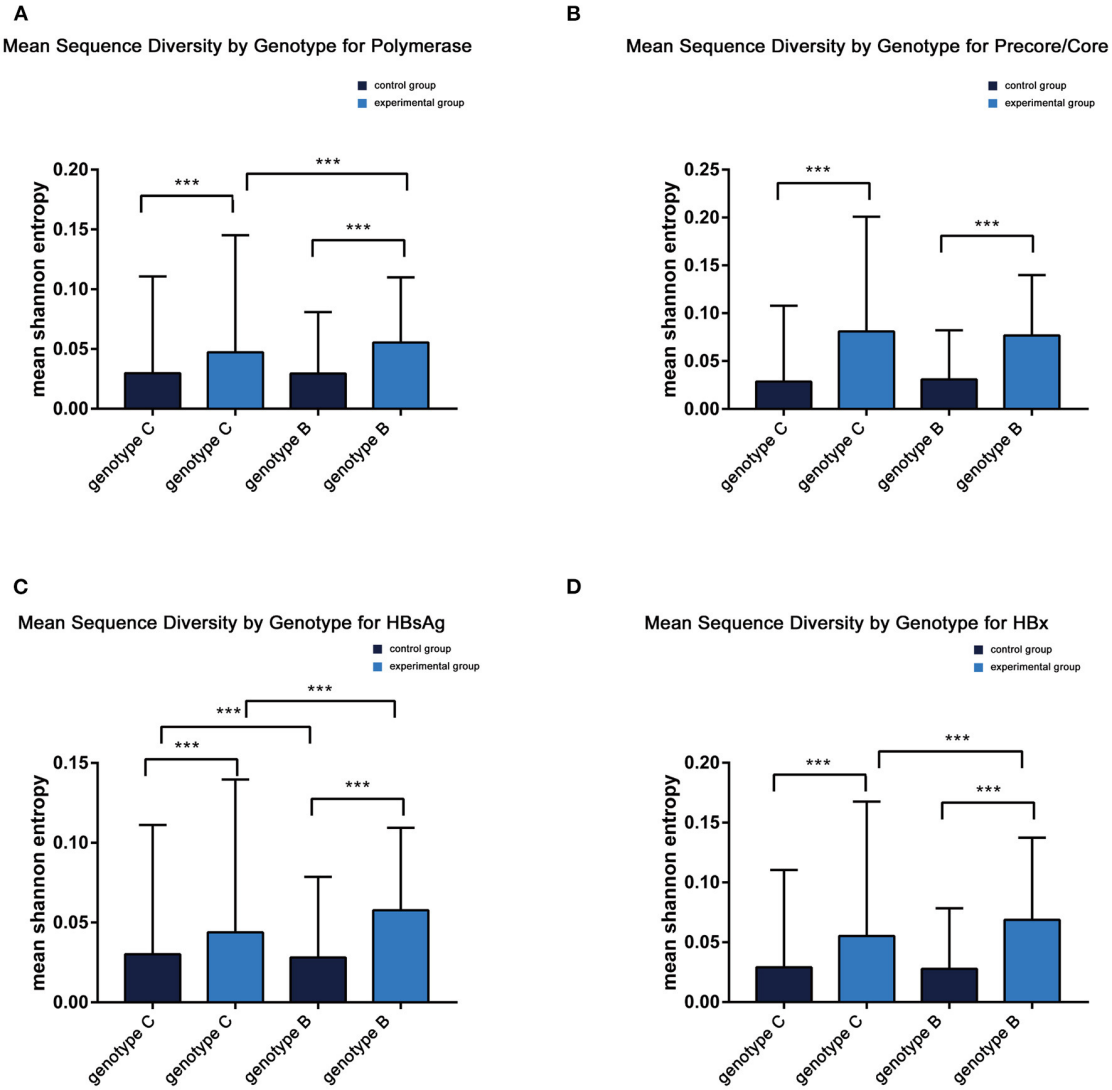
**(A–D)** Mean sequence diversity across genotypes among baseline sequences identified by Shannon entropy in different regions of the HBV genome. Error bars indicate SEM; *** indicates *p* < 0.001. HBx, hepatitis B X; HBsAg.

Comparable sequence diversity was found among the full-length HBV genomes in RA CHB (Figure 2) and four ORFs of HBV genotypes B and C (Figure 3). The Shannon entropy (Sn) values of nucleotides for the full-length HBV genome and the P/C/S/X genes encoding LHBsAg, MHBsAg, HBsAg, HBxAg, HBcAg, Pol, reverse transcriptase (RT), and the core promoter (CP) region were significantly greater in the experimental group than in the control group. The sequence distribution diversity of the P/C/S/X regions was significantly different between the experimental and control group according to Shannon entropy analysis by R code (Figure 3). Compared to the control group, the experimental group exhibited significantly higher Shannon entropy values (*p* < 0.05) at both the nucleotide and amino acid levels. Different from the control group of HCC patients, the sequences diversity of the P and X regions was significantly different in the experimental group, regardless of genotype B or C. The control group typically exhibited fewer mutations than did the experimental group, with an exceptional number of mutations found in the genes encoding LHBsAg, MHBsAg, and HBsAg in genotypes B and C, located in HLA I T cell epitopes or multiple types of epitopes (Figure 2).

### Selective Pressure

**Figure 4:**
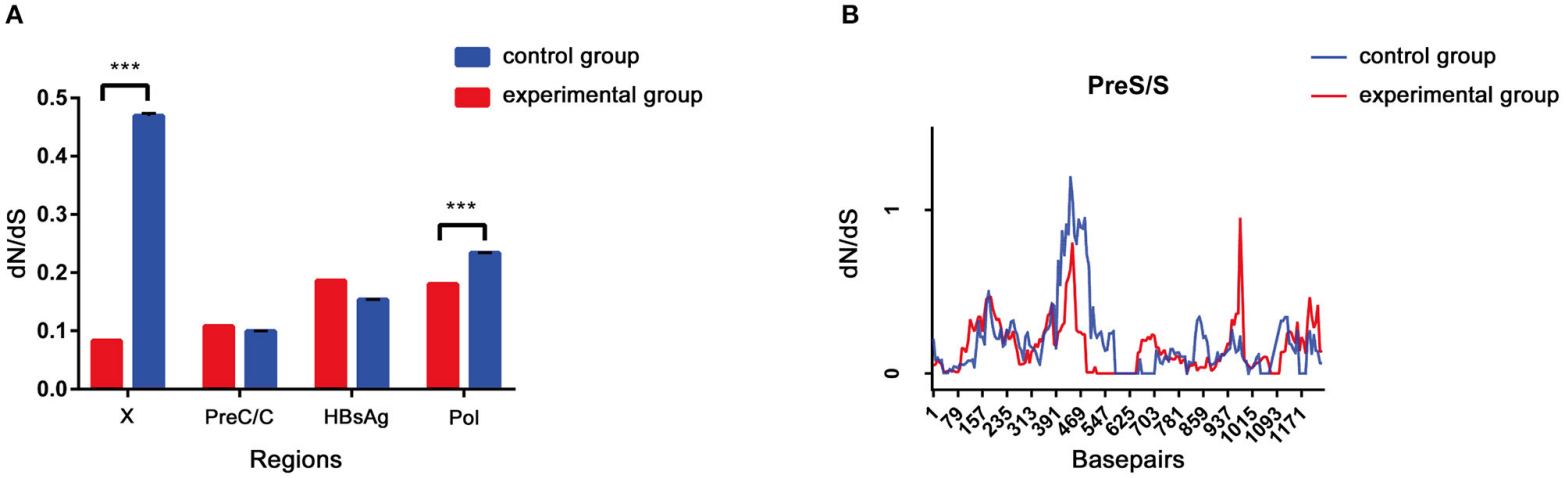
**(A,B)** The dN/dS of the S gene was verified by a sliding window analysis using the KaKs calculator (window length, 57 nt; window step, 6 nt).

The dN/dS ratio was used to measure selective pressure across individual lineages established with 28 experimental group sequences and control group sequences. The non-synonymous/synonymous rate ratio (ω = dN/dS), shows selective pressure at the protein level. If dN < dS (then ω < 1) is calculated, purifying selection will reduce their fixation rate. If dN > dS is favored by Darwinian selection, they will be fixed at a higher rate so that ω > 1 and is considered as adaptive protein evolution. We utilized the free ratio model (M1) in PAML4 to calculate the dN/dS ratio for four protein-coding genes (Pol, LHBsAg, HBxAg, and PreC/C) individually, and the variability of dN/dS in the pre-S/S region was further detailed using sliding window analysis (Figure 4). Overall, the four protein-coding genes in the two study groups exhibited negative selection and purifying selection reduced their fixation rate such that dN/dS < 1. However, the dN/dS ratio of the P/X regions was significantly higher in the control group than in the experimental group (0.1802 ± 0.0003, *p* < 0.001; 0.0827 ± 0.0011, *p* < 0.001). In constrast, the dN/dS values of the pre-S/S region showed no significant difference between the experimental group and control group (0.1859 ± 0.0006 vs. 0.1534 ± 0.0004, *p* = 0.06). The sequence mutations between the P and X regions are related to HCC progression, and the study group may have a low risk. Moreover, based on dN/dS values in pre-S/S region, more positive selection between nucleotide positions 313 and 547 occurred in the control group, whereas significant selection pressure between nucleotide positions 859 and 1,015 occurred in the experimental group. Therefore, it seems that the pre-S/S sequences of experimental group will evolve similar to those of the control group, associated with the risk of further HCC progression.

### Positive Selection

A codon-based molecular evolution model was used to identify positive selection in the HBsAg protein-coding gene. In both groups, HBsAg was under positive selection according to the M8 model. In the experimental group, LHBsAg, MHBsAg, and HBsAg were all under positive selection according to the M8 model. Seven positively selected sites were identified in pre-S1, five in pre-S2, and four in S using the empirical Bayes method (Table 1). These sites of positive selection among pre-S/S regions improved the adaptability of HBV and were conducive to its survival.

**Table 1:** Codons in the HBV pre-S/S regions under positive selection pressure.

**Region**	**Codon(pre-S/S)**	**Posterior probability**	**Omega (±s.e.m.)**
pre-S1	47 L	1.000**	4.221 ± 0.830
	60 A	0.992**	4.208 ± 1.029
	62 A	0.955*	3.984 ± 0.575
	73 G	0.984*	3.402 ± 0.592
	84 I	0.978*	3.435 ± 0.525
	90 A	0.969*	4.132 ± 1.120
	128 H	0.979*	3.959 ± 1.284
pre-S2	135 R	0.963*	3.646 ± 1.472
	138 Q	0.953*	4.217 ± 0.838
	139 L	0.950*	4.027 ± 1.230
	141 P	1.000**	4.221 ± 0.830
	177 N	0.977*	4.159 ± 1.089
S	374 Y	0.998**	4.219 ± 0.835
	387 M	0.988*	4.206 ± 0.857
	221 T	0.991**	3.441 ± 0.512
	300 I	0.957*	3.324 ± 0.717

Positively selected sites based on naive empirical Bayes (NEB) analysis (*p > 95%; **p > 99%). ω > 1, means that significantly positive selection is for adaptive protein evolution.

### Homologous Modeling Analysis of Positive Selection in HBsAg

**Figure 5:**
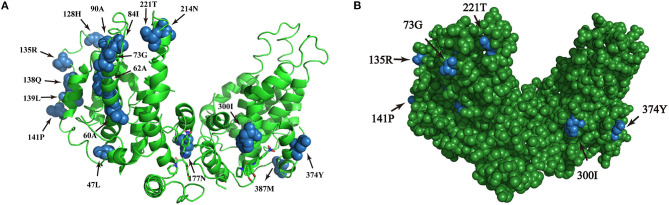
**(A,B)** Homologous modeling analysis of sites of positive selection in HBsAg. The blue highlights the presence of point mutations within HBsAg and the green shows the HBsAg structure.

The amino acid sequence variations of HBsAg were modeled by using PyMOL software. Six sites of positive selection found in HBsAg are mainly located in antigen epitopes (Figure 5).

## Discussion

The coexistence of lower levels of HBsAg and higher levels of HBV DNA was a unique serological profile identified in this study. Low replication activity was observed in the tumors of HCC patients, due to the low HBV pgRNA-to-DNA ratio, similar to the control group. Previous studies have shown that the levels of HBsAg correlate with HBV DNA and intrahepatic covalently closed circular DNA (cccDNA) levels. However, some studies have found that serum HBsAg levels correlate weakly or moderately with serum HBV DNA and are weakly or not associated with intrahepatic cccDNA. Low HBsAg can predict subsequent HBsAg loss and the risk of HCC (18). The combination of HBsAg and HBV DNA suppression is now considered as the most important endpoint for clinical trials and the ultimate short-term goal of treatments with the medications currently available (19). Thus, chronic HBsAg carriers may simultaneously experience a lowering of the viral load to 2,000 IU/ml and the HBsAg level to 100 IU/ml, reducing the risk of liver cancer (20). The purpose of our study was to investigate whether CHB patients in the LR/RA phase with special serological profiles require monitoring management.

The natural history of HBV infection can generally be divided into four periods: the immune tolerance phase, immune clearance phase, inactive phase and reactivation phase. Previous studies have suggested that patients with inactive HBV infections have a lower risk of developing liver cancer because they exhibit lower levels of HBsAg and there is a probability of spontaneous clearance (21). In addition, a quantitative HBsAg level < 100 IU/ml can be used as the optimal threshold for predictive clearance (22), but no consensus regarding the exact value of the reference endpoint as an alternative threshold in clinical treatment has been reached (23). Some studies have shown that most patients with low-level viremia (LLV; <2,000 IU/mL) in the inactive phase of CHB infection present minimal evidence of liver injury (24). However, we focused on a CHB cohort with high-level viremia but low-level HBsAg in this study. An undetectable viral level rather than loss of HBsAg is associated with a lower risk of HCC (25). Determination of whether the elimination of HBsAg as the endpoint of long-term antiviral treatment reduces, or merely delays the occurrence of liver cancer will require the discovery of more biomarkers to support the explanation. A unique feature of LR/RA CHB observed in this study was that there was a possibility of immune escape.

Moreover, HBV whole-genome sequences showed greater diversity in LR/RA CHB with lower levels of HBsAg and higher levels of HBV DNA than in control group. Additionally, the mean Shannon entropy score was not different between genotype C and genotype B subjects. Although we did not identify any hot-spot mutations or pre-S deletions associated with HCC, we found a higher frequency of P/S/X/pre-C/C mutations in LR/RA CHB based on the mean Shannon entropy. Previous studies have shown that mutation in the pre-S/S region affects HBsAg expression, with the final manifestation being the immune response between the virus and host; that is, the stronger is the immune response, the lower is the HBsAg level (26). The low serum level of HBsAg observed in HBsAg-negative CHB (<100 IU/mL) suggests a high probability of negative HBsAg conversion (27); on the other hand, a negative level of HBsAg serology suggests that the virus integrates into the host genome to some degree. This finding was consistent with the higher nucleotide Sn (frequency of mutated positions) and dN values for these genes in the experimental group (*p* < 0.001) (Figure 3). Previous studies have suggested that increased diversity over the entire HBV genome at baseline is associated with reduced HBsAg loss under NAs therapy in patients with genotype A and D, but our experimental group consisted of genotype B and C patients with LR/RA CHB who did not receive any NAs therapy. Regardless, the mechanism underlying the impact of whole-genome diversity on low HBsAg and high viral replication in LR/RA CHB remains unclear.

The dN/dS ratio is used to measure the strength of selection acting on protein-coding genes. To estimate the immune selection pressure on the HBV genome, we calculated the dN/dS ratios of protein-coding genes. The dN/dS ratios of two genes (HBxAg and polymerase) were significantly lower in the experimental group than in the HCC group (*p* < 0.001), indicating relatively strong negative selection in the former compared to the latter. However, the dN/dS ratios of the other two genes (HBsAg and pre-C/C) in the experimental group were not significantly different from those in the HCC group. In CHB, the frequency of mutations (or deletions) in the pre-S/S region caused by natural selection or antiviral therapy is very high (28). More commonly, in liver cancer tissue samples (29), pre-S mutations increase the incidence of HCC by 377-fold (30), which has been confirmed in several prospective cohort studies (31). In addition, S region mutations are associated with liver fibrosis and liver cancer due to HBV RNA splicing (32). Subsequently, the dN/dS of the pre-S/S region was verified and the results were in agree with a previous study reporting that HCC patients have different regions of positive selection due to the special mode of CHB. This may result in the risk of HCC in the experimental group and needs further study in the future.

Due to the special phenotype of the patients in this study, we analyzed the codons of the HBV pre-S/S region that were under positive selection. Seven codons under positive selection were found in pre-S1, five in pre-S2, and four in S, which suggests that accumulating mutations may provide an opportunity for the virus to escape from host immune pressure. The expression level of HBsAg in RA CHB is usually low, but there are many factors influencing this phenotype, including non-specific laboratory detection caused by viral gene mutations. In addition, the “α” determinant (amino acid residues 124-147) of the S region is an epitope recognized by the antibody, and point mutations in this determinant will result in a low affinity of the corresponding antibody (33). In our study, five sites of positive selection with statistical significance were found in the “α” determinant (128H/135Q/135R/139L/141P), showing evolutionary diversity of HBV in the experimental group, which would be conducive to the survival of the virus. The combination of multiple mutations in the HBV pre-S/S region can also affect viral immunogenicity, along with the methodological limitations of existing commercial HBsAg quantification reagents (34), often delaying or interfering with clinical diagnosis and treatment. We further speculate that the pre-S/S mutant strains may lead to disproportionate synthesis and secretion of the virus such that the serum HBsAg titer does not reflect the viral replication ability (26). The preliminary data from this study also indicated that the HBV genome sequence in LR/RA CHB exhibits more sites of positive selection in the pre-S/S region, and we must further study the interaction mechanism between the virus and host. As pre-S/S region mutations have been confirmed to be associated with HCC, we still need to evaluate the risk of pre-S/S mutant strains for the progression of disease.

It is worth mentioning that the present study is the first to describe the genome characteristics of the special mode with coexisting low levels of HBsAg and high levels of HBV DNA. However, our study has a few limitations. First, the case number was limited, and further large-scale studies are needed to confirm the results. Second, as this study used HCC sequences from databases as reference, clinical information was not obtained. Hopefully, the sites of positive selection identified in the pre-S/S region showing significant differences can be used as candidate molecular markers, though further mechanistic studies are still required.

## Conclusion

We speculate that increased positive selective pressure on the pre-S/S region of HBV may allow immune escape and lead to possible alterations in HBV serotype. Understanding the evolution of HBV in patients with LR/RA CHB with a high viral load can increase our understanding of the pathogenesis of CHB.

## Data Availability Statement

The raw data supporting the conclusions of this article will be made available by the authors, without undue reservation.

## Ethics Statement

The study was approved by the Ethics Committee of Fourth Affiliated Hospital of Zhejiang University School of Medicine.

## Author Contributions

ZZ performed the sample collection, clonal sequencing and analysis, data interpretation, and drafted the manuscript. JW assisted with the clonal sequencing. WB assisted with the sequencing and data analysis. XX and WX assisted with recruitment of RA CHB patients and clinical data collection. YW was responsible for the study design and data interpretation, and was a major contributor to the manuscript editing and critical revision of the article. All authors read and approved the final manuscript.

## Conflict of Interest

The authors declare that the research was conducted in the absence of any commercial or financial relationships that could be construed as a potential conflict of interest.
