# Supplementary material for: Evolutionary Analysis of Pre-S/S Mutations in HBeAg-Negative Chronic Hepatitis B With HBsAg < 100 IU/ml
Source: Front Public Health. 2021 Apr 26;9:633792. doi: 10.3389/fpubh.2021.633792 (PMC8107265; doi:10.3389/fpubh.2021.633792)
Supplement: Supplementary file 1 [file Data_Sheet_1.docx]

**Table S1. Characteristics of the Study Cohort. Data are n (%) and median (IQR). ALT, alanine aminotransferase; AST, Aspartate aminotransferase; HBsAg, hepatitis B surface antigen.**

| **Characteristics** | **Total(n=28)** |
| --- | --- |
| Sex, Male(%) | 49(69.01) |
| Age, years, median (IQR) (range) | 46(23-82) |
| ALT, U/L, median (IQR) (range) | 29(8-48) |
| AST, U/L, median (IQR) (range) | 26(17-47) |
| HBsAg, IU/mL, median (IQR) (range) | 32.85(0-88.37) |
| HBV DNA, LogIU/mL, median (IQR) (range) | 3.71(3.30-7.25) |
| HBV DNA，>20000 IU/ML(%) | 42.25 |

**Table S2. Accession numbers of control group in NCBI.**

| **Group** | **Accession numbers** |
| --- | --- |
| **Sequences of Control group** | EU522069, AF182805, AB014368, EF137802, AB014360, AB014367, AB670237, AY206374, JQ429081, JQ429080, JQ027328, JQ027334, EU660230, AY206380, AB014366, AY206377, EU919174, EU522073, AY206375, AY206373, EU919161, JQ027329, EU881997, AY206383, JQ027315, EU564822, EU882003, EU919175, AY206391, EU882001, EU919170, EU919172, EU487257, EU564825, JQ027331, JQ027330, EU522067, AY206390, JQ027325, AY206387, EU487256, AB014365, JQ027314, JQ027316, AY163870 |
